# Rationales for the Use of Cancer Stem Cells Markers in the Staging of Papillary Thyroid Carcinoma

**DOI:** 10.1155/2019/1659654

**Published:** 2019-06-24

**Authors:** Noah Abd-Alkader Mahmood, Amer Talib Tawfeeq, Israa Mhdi Al-Sudani, Zaynab Saad Abd-Alghni

**Affiliations:** ^1^Molecular Biology Lab, Department of Molecular Biology, Iraqi Center for Cancer and Medical Genetics Research, Mustansiriyah University, Baghdad, Iraq; ^2^College of Medicine, Ibn Sina University of Medical and Pharmaceutical Sciences, Baghdad, Iraq

## Abstract

Fine needle aspiration biopsy (FNAB) is a standard procedure for the detection of thyroid nodules malignancy, yet 10-25% of the sample diagnosed may go undetermined or suspicious. The utility of cancer stem cell markers (CSCM) as a differential diagnosis molecular marker in nodules of suspicious decision in FNAB was hypothesized. Papillary thyroid carcinoma (PTC) and thyroid fibroadenoma (TFA) samples were selected to test the hypothesis. The samples employed in this study were from patients who had thyroid hyperplasia and a suspicious or undetermined diagnosis by FNAB. The patient underwent a successful thyroidectomy at Al-Yarmouk Teaching Hospital in Baghdad between January 2015 and December 2017. All nodule samples underwent a systematic histopathological examination after resection. Tumors diagnosed as PTC and those diagnosed as fibroadenoma (TFA) were selected for this study. Collectively 39 PTC and 11 TFA nodules were included. Quantitative reverse transcriptase real-time PCR (qRT-PCR) and immunohistochemistry (IHC) were used to determine levels of mRNA and proteins of CSCM ALDH1A1, CD44, ABCG2, and Oct3/4 in both types of tumors were used. This study revealed that the expression levels of CSCM were significantly increased in PTC tissues when compared to benign tissues and the positive correlation was found between the CSCM expression levels and tumor stage, size, and gender. In conclusion, for a more precise diagnosis, we suggest these markers be included in what is currently available to characterize malignancy from what is not in thyroid cancer, as well as for the staging process of PTC.

## 1. Introduction

Thyroid carcinoma is the fifth most frequent cancer diagnosed in the United States and eighth diagnosed in Iraqi women, and its incidence in Iraqi women has noticeably increased [1, 2]. The frequency of thyroid cancer has increased in women between 20 and 55 years old, and it is a widespread cancer type in children who are exposed to ionizing radiation in treating head and neck cancers [3]. Among anaplastic thyroid carcinoma (ATC) and follicle thyroid carcinoma (FTC), papillary thyroid carcinoma (PTC) is considered the most common thyroid malignancy [4]. The recent consensus in cancer biology suggests that a tumor could be formed through two different models [5]. In the first model, tumor cells can develop as a result of the accumulation of genetic mutations and genetic instability, leading to a process of cellular clonal selection for specific traits favorable to the tumor environment [6, 7]. The other model hypothesized that the cancer cells develop as progenies of a small subpopulation of cancerous cells called cancer stem cells (CSCs) [7]. These cells have the capacity for self-renewal and multilineage potential [8]. These two models are not mutually exclusive in the explanation of the cellular heterogeneity of cancers [9]. However, conducting the CSC model can clarify some dilemmas in cancer biology including tumor recurrence, metastasis, and resistance to conventional radio-chemotherapies [6]. The concept that cancer might develop from a small population of cells termed CSCs came from earlier studies of hematopoietic malignancies and it was first recognized in solid tumors by Al-Hajj and colleagues. They were able to detect expression of some normal stem cell markers in a small population of cells isolated from the primary tumor of breast cancer patients; these cells were highly tumorigenic [10, 11]. Afterward, the identification and characterization of such tumorigenic subpopulation harboring these markers in other types of tumors were recognized. Cancer stem cells expressing such biomarkers were studied extensively in many tumor types to understand the behavior of these cells in cancer progression, metastasis, and resistance to conventional therapies [12, 13]. Some of these markers were recognized on the cell membrane, and some were predominantly expressed in the cytoplasm or in the nuclei of normal stem cells and CSCs. For thyroid tumors, these markers include CD44, ALDH1A1, OCT3/4, and ABCG2. Fine needle aspiration biopsy (FNAB) is mainly used in the diagnosis of thyroid nodules; this diagnostic tool importantly can reduce thyroid resection surgical operations by a decisive differential diagnosis of all types of thyroid nodules. This test influences subsequent clinical decisions for the patients; however while using this test, a false negative diagnosis cannot be excluded [14]. In reality, challenges remain in using the FNAB test to diagnose thyroid cancer, these challenges associated with what known as indeterminate cytology. In the United States, about 20-30% of the thyroids nodules diagnosed each year that underwent surgical thyroid resection are found to be benign fibroadenoma. This percentage was constant for the last two decades [15]. Other molecular tests such as* BRAF* and* K-RAS* oncogenes mutation, detection of genetic rearrangements for* RET/PTC* and* PAX8/PPARγ*, microRNA profiles, and aberrant methylation of important genes were proposed to make the differential diagnosis more efficient [16]. In this research, we have a select number of PTC tumors and TFA nodules that were indicated for surgery after being diagnosed with FNAB as suspiciously malignant or having undetermined features. All selected samples were diagnosed and staged histopathologically after surgery. We asked, to what extent the level of cancer stem cell markers could contribute to the staging of PTC, and the relationship between these markers and patients' clinical features, and could these markers be used to differentiate between PTC and TFA in thyroid cancer that is suspicious or undetermined by FNAB.

## 2. Patients and Methods

### 2.1. Patients and Ethics

All patients enrolled in this study were admitted to Al-Yarmouk Teaching Hospital in Baghdad between January 2015 and December 2017. They were informed of the study purpose and provided with a written informed consent form. The study was approved by the Ministry of Health and Ministry of Higher Education and Scientific Research ethical committees represented by Al-Yarmouk Teaching Hospital ethical committee and the ethical committee of the Iraqi Center for Cancer and Medical Genetics Research (both are IRB). The patient candidates were 41 females and 9 males, with a mean age of 37 years (23-55 years). All the patients enrolled in this study had not received chemo- or radiation therapy.

### 2.2. Tumor Characteristics

According to the standard protocols, all hospitalized patients were diagnosed with thyroid hyperplasia initially using palpation, ultrasound, and FNAB [17]. They indicated for surgery after having definite or suspicious and/or undetermined diagnosis for thyroid cancer. All patients who had suspicious and/or undetermined diagnoses were selected for this study and those with defined diagnoses were excluded. After surgery, histopathological examination was carried out to define the diagnosis of all cases according to the World Health Organization endocrine glands tumor classification system [18]. Of that selection, 39 tumors of papillary thyroid carcinoma (this type was the majority of tumors available during this study) as well as 11 benign tumors (TFA) were harvested from that patients set. Fresh thyroid tumors tissues was divided into two parts after it was collected: one part was fixed in formalin and embedded in paraffin to be used in the histopathological examination and immunohistochemistry staining for the determination of tumor stage, type, and cancer stem cell marker. The other part was stored in −80°C to subsequent use for RNA extraction.

### 2.3. RNA Extraction

To determine the mRNA levels of* ALDH1A1*,* CD44*,* OCT3/4*, and* ABCG2*, total RNA was extracted from PTC tissues and the benign tissues using ACCUZOL™ (Bioneer, Sangam-dong, Mapo-Gu, Seoul, 121-835, Republic of Korea) total RNA extraction reagent according to manufacturer instructions.

### 2.4. Real-Time PCR

The mRNA levels of* ALDH1A1*,* CD44*,* OCT3/4*, and* ABCG2* in PTC and benign tumors were determined by quantitative real-time PCR. The relative gene expression and 2^−∆∆ct^ method was used. ß-actin mRNA was used as a normalizer and benign fibroadenoma mRNAs as a calibrator [19, 20]. Forward and reverse primers for each gene were designed using the NCBI (National Center for Biotechnology Information) database. The accession number and the primers sequences of each gene are shown in Table 1. The Primer-Blast tool from the NCBI website (https://www.ncbi.nlm.nih.gov/nucleotide) and UCSC in-silico PCR tool (https://genome.ucsc.edu) were used to verify all primers. The primers were also verified during RT-qPCR by the formation of single-peaked disassociation curves. Results were obtained from three replicates and expressed as the mean ± standard error (SE) of three independent experiments.

The PCR amplification reaction was carried out using KAPA SYBR fast universal one step qPCR kit (KABA Biosystems, Boston, MA, USA). The Stratagene MxPro 3005P system was employed in the study (Agilent Technology, Los Angeles, CA, USA). The reaction was performed in a 20 *μ*l volume and the thermal profile was 5 minutes at 42°C for 1 cycle for cDNA synthesis, 3 minutes at 95°C for 1 cycle, followed by 40 cycles at 94°C for 15 seconds, 55°C for 30 seconds, and 72°C for 30 seconds.

### 2.5. Immunostaining and Scoring

Protein expression of ALDH1A1, CD44, OCT3/4, and ABCG2 was evaluated immunohistochemically. Four millimeters of the paraffin-embedded section was cut and mounted on positive charge glass slides and deparaffinized in xylene and rehydrated by a series of ethanol solution. The slides were incubated with primary antibodies: anti-CD44 (sc-9960 L, Lot# E0813 Santa Cruz Biotechnology, California, USA), anti-OCT3/4 antibody (sc 5279 Santa Cruz Biotechnology, California, USA), anti-ABCG2 (B2738A United States Biological, Salem, MA, USA), and anti-ALDH1A1 antibody (A1334-33W United States Biological, Salem, MA, USA) overnight at 4°C. Slides were incubated with horseradish Peroxidase conjugate detection reagent and with complex avidin-biotin for 30 minutes at room temperature. The slides were visualized using diaminobenzidine (DAB) (ImmunoCruz LSAB staining system, sc-2050, Santa Cruz Biotechnology, California, USA). After that, the tissues were stained with hematoxylin as a counterstain, dehydrated with graduated ethanol and then with xylene. Finally, the slides were mounted with a water-free mounting medium (DPX) and then analyzed by a light microscope at (400x). The negative control was treated with the same steps above except the incubation with the primary antibody. Evaluation of immunohistochemical staining was carried out with a blind to the patient's data and pathological features. The percentage and intensity of the staining were considered in this study. Normal cells that present in the whole tissue were scored as negative 0 percentage (no positive staining), score 1: (1-10% positive tumor cells), score 2: (11-50% positive tumor cells), and score 3 (51-100% positive tumor cells). The staining intensity was considered as 0 (no staining), 1(+) (weak positive staining = yellow), 2 (++) (moderate positive staining= yellow-brown), and 3 (+++) (strong positive staining = brown). Both 0 and score 1 were considered as low expression and score 2 and score 3 were considered as high expression. Expression of each gene over 10% was considered positive [21, 22].

### 2.6. Statistical Analysis

Significance in the levels of gene expression between thyroid papillary carcinoma stages and benign fibroadenoma was calculated using SAS (Statistical Analysis System, version 9.1). A one-way ANOVA and least significant difference (LSD) post hoc test were performed to assess significant differences among means [23]. A chi-square test for independence was used to assess the association between the categories. The correlation coefficient between PTC and tumor staging was calculated.* P*< 0.05 was considered statistically significant.

## 3. Results

The systematic histopathological staging of each sample after surgery revealed that, among PTC samples selected for this study, 25 (64%) of the samples were of stage I, 8 (21%) were of stage II, and 6 (15%) were of stage III. The four genes (*ALDH1A1*,* CD44*,* OCT3*/*4*, and* ABCG2*) chosen to detect their expression levels in all 39 samples of PTC as well as in 11 thyroid fibroadenoma samples were selected for their potential role as cancer stem cell markers in thyroid cancer and their potential importance in thyroid cancer initiation and progression [24–26]. As our results showed, expression levels of* ALDH1A1*,* CD44*,* OCT3/4*, and* ABCG2* genes were relevant to papillary thyroid cancer stages starting from stage I to stage III, whereas in the benign tumor it was not. Expression levels of* ALDH1A1*,* CD44*,* OCT3*/*4*, and* ABCG2* gene were 9.78, 7.02, 9.75, and 7.96 times more in stage III thyroid carcinoma than that in benign thyroid mass, respectively (Table 2).

### 3.1. Protein Expression Levels of Cancer Stem Cell Markers as Determined by Immunohistochemistry

**Figure 1:**
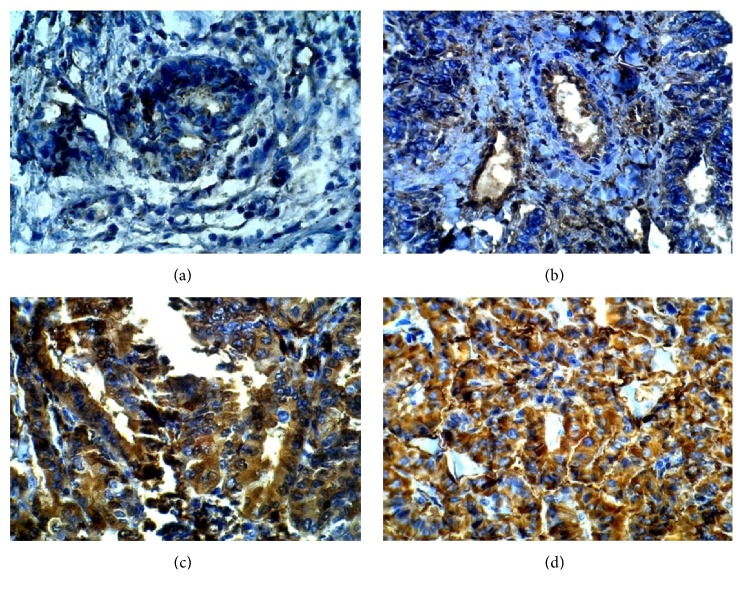
Representative image of immunohistochemistry staining of ALDH1A1 in benign tumor tissue and in papillary thyroid carcinoma tissues (400X). (a) Benign tumor section. (b) Stage I papillary thyroid cancer tissue. (c) Stage II papillary thyroid cancer tissue. (d) Stage III papillary thyroid cancer tissue.

**Figure 2:**
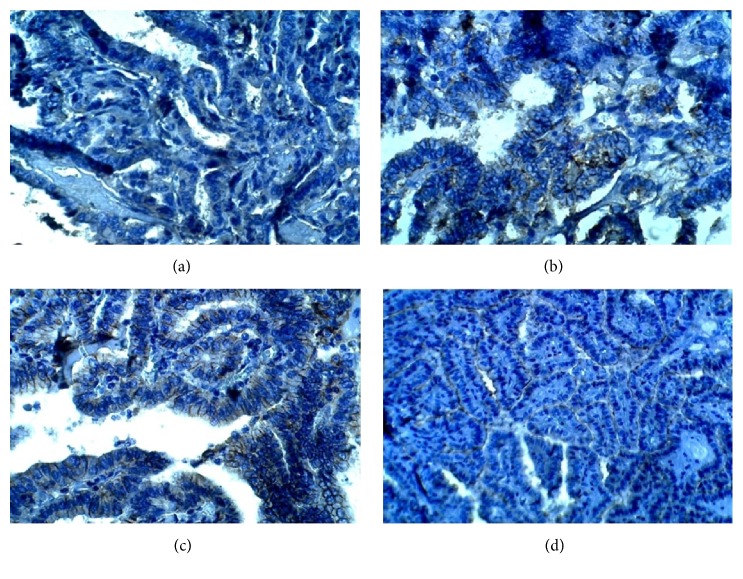
Representative image of immunohistochemistry staining of CD44 in benign and papillary thyroid carcinoma tumor tissues (400X). (a) Benign tumor section. (b) Stage 1 papillary thyroid cancer tissue. (c) Stage II papillary thyroid carcinoma. (d) Stage III papillary thyroid carcinoma.

**Figure 3:**
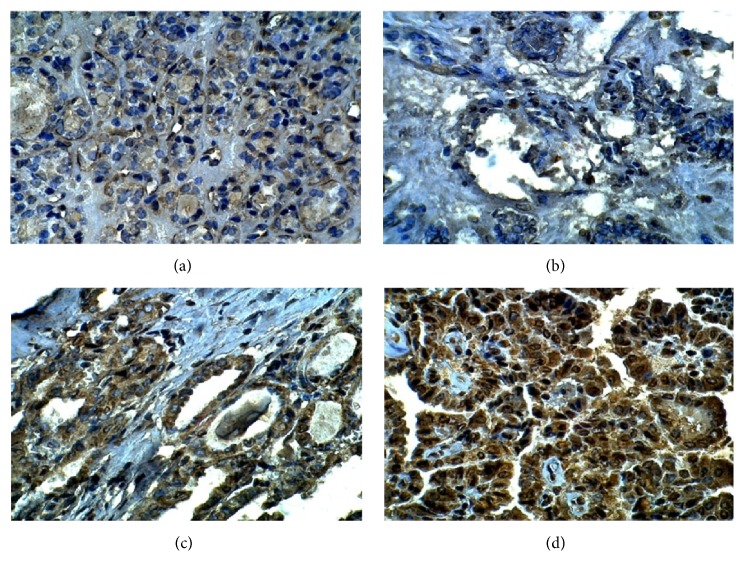
Representative image of immunohistochemistry staining of OCT3/4 in benign tumor tissue and papillary thyroid carcinoma (400X). (a) Benign tumor section. (b) Stage I papillary thyroid cancer tissue. (c) Stage II papillary thyroid cancer. (d) Stage III papillary thyroid cancer.

**Figure 4:**
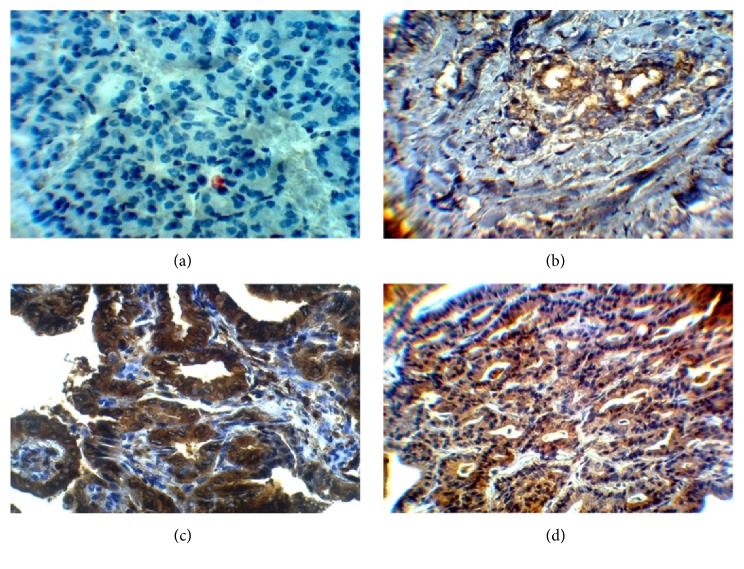
Representative image of immunostaining of ABCG2 in benign tissues and papillary thyroid carcinoma tissues (400X). (a) Benign tumor tissue. (b) Stage I papillary thyroid cancer tissue. (c) Stage II papillary thyroid cancer. (d) Stage III papillary thyroid cancer.

Immunohistochemical staining was performed to determine the expression patterns of ALDH1A1, CD44, OCT3/4, and ABCG2 in the 39 PTC as well as in the 11 TFA tissues. Immunostaining showed that ALDH1A1 positive was expressed in 27 out of 39 (79%) papillary thyroid carcinoma tissues (P=0.0001) (Table 3 and Figure 1). CD44 immunostaining showed that 24 out of 39 papillary thyroid carcinoma tissues have a high positive expression of CD44 (P=0.003). The overall positive ratio was 61.5%. Approximately 38.5% of papillary thyroid carcinoma tissues showed low CD44 expression (Table 3 and Figure 2). On the other hand, OCT3/4 was expressed in the nucleus of the cells. Immunostaining showed that positive expression of OCT3/4 was found in 25 out of 39 thyroid cancer tissues (P=0.001). The overall positive ratio was 64%. Approximately 36% of thyroid cancer tissues showed low OCT3/4 expression (Table 3 and Figure 3). For ABCG2, the result showed that the positive expression of ABCG2 was found in 25 out of 39 cancer tissues (P=0.003). The ABCG2 protein was positively expressed in 64% of cancer tissues compared to the 36% low ABCG2 expression level (Table 3 and Figure 4).

### 3.2. Correlation between Cancer Stem Cell Markers and Thyroid Tumor Staging

**Figure 5:**
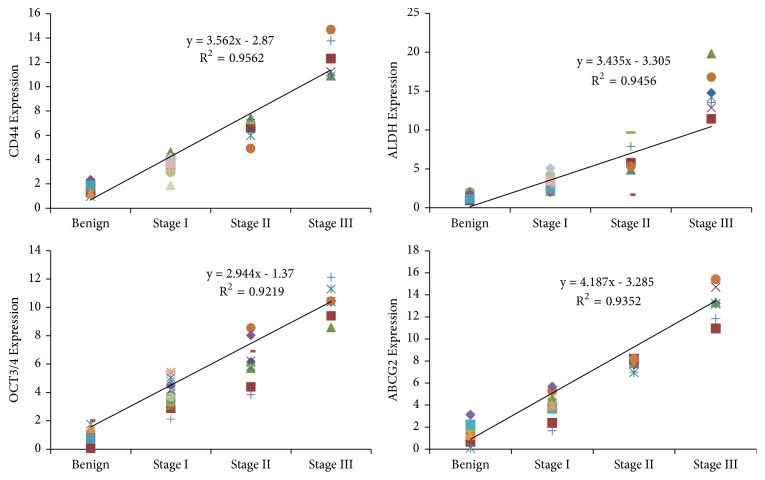
Correlation coefficient between levels of cancer stem cells markers expressed in papillary thyroid cancer samples and benign tumor with expression levels of cancer stem cells markers.

The correlation coefficient between the expression level of cancer stem cell markers ALDH1A1, CD44, OCT3/4, and ABCG2 with the stages of PTC tumors as well as TFA samples was calculated. Results indicated a strong positive correlation between pathological stages of PTC and levels of cancer stem cell markers (Figure 5)

### 3.3. The Relationship between Cancer Stem Cells Expression Levels and Clinicopathological of Thyroid Cancer

The expression levels of cancer stem cell markers ALDH1A1, CD44, OCT3/4, and ABCG2 with clinical-pathological features of patients with papillary thyroid carcinoma are summarized in Table 4. The results showed that the expression level of ALDH1A1 was correlated with gender, tumor size, and tumor stage (*P*=0.001, 0.02, and 0.01, respectively). The same results were found in the correlation between the expression level of OCT3/4 and gender, tumor size, and tumor stages (*P*=0.013, 0.043, and 0.015, respectively). Results showed a negative correlation between the expression level of OCT3/4 and the patient's age. On the other hand, the expression level of ABCG2 was correlated with gender, tumor size, and tumor stage (*P*=0.02, 0.04, and 0.001, respectively). No significant positive correlation was found between the expression level of ABCG2 and the patient's age. The expression level of CD44 was also significantly correlated with tumor size, tumor stage, and gender (*P*=0.001, 0.035, and 0.05 respectively). There was no significant variation between the expression levels of CD44 and patients age (Table 4).

## 4. Discussion

Diagnosis of thyroid cancer is a multistep process, starting with the physical examination of the thyroid gland area and ending with the FNAB through several blood tests, sonography, and other imaging tests such as computed tomography (CT) and positron emission tomography (PET) [27]. Efforts to find new methods to precisely determine a diagnosis for this cancer are continuous [27, 28]. In this respect, molecular biology methods represent the best choice to make the long road shorter. These methods employ the detection of the driver mutations in specific oncogenes to confirm the malignancy in biopsies of these tumors [29]. However, these tests do not give an idea about the prognosis of this disease. In accordance with these efforts, we suggest employing the levels of cancer stem cell markers not only as a manifestation of malignancy but also as a tool to detect the extent of tumor burden. The affirmative presence of cancer stem cells and their certain related markers in almost all types of solid tumors paved the way to the exploitation of these markers in the determination of pathogenesis and prognosis of these cancers. The level of cancer stem cell marker expression in different types of tumors is yet to be employed in the diagnosis of different types of cancer [30].

To our knowledge, this is the first study to explore the relationship between the expression levels of CSC markers and the clinical features of Iraqi patients with papillary thyroid carcinoma. The research question had two branches: first, to what extent we can employ these markers in differentiating between papillary thyroid carcinoma and benign thyroid adenoma in cases not well determined and suspiciously diagnosed by FNAB. Second, can cancer stem cell markers serve the process of PTC staging. All the suspicious cases in FNAB of thyroid were indicated for surgery; of those, we selected PTC (since PTC was the majority of cases that emerged among other types of thyroid cancer during the time of the study) and TFA to carry out a comparison. All cases selected were histopathologically staged and we started to determined levels of cancer stem cell markers in each sample of each staged tumor. Our results indicated statistically significant elevated levels for these cancer stem cell markers in all PTC tumors tested compared to TFA samples. In this regard, we may suggest the inclusion of these features in the differential diagnosis of PTC and TFA. Although we have used tissue masses to perform the test and conclude this suggestion, it would be very useful to have a conspectus indication in the determination of precise malignancy decision if implemented to FNAB samples.

There was some evidence that cancer stem cell markers could be detected in the benign tumors of endocrine glands; therefore, cancer stem cell markers may not be associated with malignancy [31]. While this evidence was confirmed convincingly, the type of benign tumor used to detect the presence of CSC markers is rarely diagnosed with malignancy and studies have recognized the presence of so many different types of CSC markers in this benign tumor [32], whereas the types of CSC markers in other solid tumors (malignant) was demonstrated as limited types with significant cellular functions related to radiochemotherapy resistance and tumor recurrence [33].

In our results, histopathological levels of the selected cancer stem cell markers did not distribute equally among stages of the PTC tumors as were detected at both phases of mRNA and protein. There was a significant difference among the three known stages of the disease: the differences followed the clinical-pathological features of this kind of cancer. The expression levels of these markers were jumped from moderate expression in the early stage to be highly expressed in late-stage through stage II papillary thyroid carcinoma as detected by immunohistochemistry and RNA. The CSC markers selected to detect in the present study have considerable significance in the pathogenesis of this cancer [34]. CD44 was first described as a cell surface marker for cancer precursor cells and exceedingly used as a reliable marker for the identification and isolation of cancer stem cells in different solid tumors [35]. Early studies interested in this glycoprotein have observed that all papillary cancers exhibit specific patterns of aberrant alternative CD44 splicing, distinguishing them from histologically normal thyroid tissue [36]. Furthermore, at that time, it was recognized that most thyroid papillary carcinomas express CD44, and it was suggested to have clinical value in confirming the diagnosis of malignancy when fine‐needle aspiration specimens were used [37]. In recent studies, the CD44 expression level was associated statistically with clinicopathologic aggressive features of PTC and was suggested to be an important factor in determining the extent of surgery [38]. Other studies showed that most papillary carcinomas express CD44 and cyclin D1, whereas it is less common in follicular neoplasm and nodular goiter, suggesting that could be helpful in diagnostically difficult cases [39]. Several studies try to identify the presence of cancer stem cells in the thyroid gland and thyroid cancer. They have recognized OCT3/4, ABCG2, and ALDH as thyroid cancer stem cell markers [40–42]. All the existing cancer stem cells or tumor initiation cells have been linked to chemotherapy resistance [14, 42].

In conclusion, the obtained data extends the research in this field in order to confirm the suggestion of using the detected cancer stem cell markers in the diagnosis (confirming malignancy) in suspicious or undetermined FNAB beside other molecular tests that have already been confirmed to be associated with thyroid cancer. According to the presented results, we suggest employing these markers in the processing of PTC tumors staged directly from FNAB.

## Figures and Tables

**Table 1:** The sequences of specific primers used for real-time qPCR.

Gene	PCR product (bp)	Primer pair sequences (5′ to 3′)	TM	Accession No.
*CD44*	96	F: CTTTGCAGGTGTATTCCACG	59.2	NM_001001389.1
		R:GCAAGGTGCTATTGAAAGCC	59.9	

*OCT3/4*	118	F:GGGTGAATGACATTTGTGGG	61	NM_203289.5
		R: CCATTCGGGATTCAAGAACC	61.6	

*ABCG2*	188	F:GATCATAGAGCCTTCCAAGC	56.6	NM_001257386
		R:CCCATCTGAGTTGATGACAG	56.5	

*ALDH1A1*	128	F:CTTGACCTCATTGAGAGTGG	55.7	NM_000689
		R:GCAATGCGCATCTCATCTGT	57	

*ß-actin*	117	F:CTCCATCATGAAGTGTGACG	57.6	NM_001101
		R:GCAGTGATCTCCTTCTGCAT	58	

**Table 2:** Relative mRNA levels of *ALDH1A1*, *CD44*, *OCT3/4*,and *ABCG2* genes in different stages of papillary thyroid carcinoma compared with benign thyroid fibroadenoma detected by quantitative real-time PCR.

Groups	*ALDH1A1*	*CD44*	*OCT3/4*	*ABCG2*
Benign (n=11)	1.51±0.13 d	1.75±0.12 d	1.10±0.16 d	1.66±0.25 d
Stage 1 (n=25)	3.15±0.19 c	3.67±0.10 c	3.90±0.23 c	4.24±0.25 c
Stage 2 (n=8)	5.71±0.82 b	6.61±0.28 b	6.15±0.50 b	7.66±0.20 b
Stage 3 (n=6)	14.77±1.23 a	12.29±0.65 a	10.73±0.52 a	13.23±0.6 a
LSD	1.518	0.716	0.9991	1.0072

A, b, c, and d symbols represent significant differences between stages for each gene at *P*> 0.05 (mean ± SE).

**Table 3:** Cancer stem cell markers protein levels in papillary thyroid tissues (n=39) and benign tumors (n=11).

	High expression N (%)	Low expression N (%)	P value
ALDH1A1			
Cancer tissues	*27 (79%)*	* 12(21%)*	* 0.0001*
Benign tissues	*2 (18%)*	* 9(82%)*	

CD44			
Cancer Tissues	*24 (61.5%)*	* 15(38.5%)*	*0.003*
Benign Tissues	*3 (27%)*	* 8(73%)*	

OCT3/4			
Cancer Tissues	*25(64%)*	* 14(36%)*	*0.001*
Benign tissues	*4(36%)*	* 7(64%)*	

ABCG2			
Cancer tissues	*25 (64%)*	* 14(36%)*	*0.003*
Benign tissues	*4(36%)*	* 7(64%)*	

**Table 4:** Relationship between cancer stem cells markers expression levels and patient's clinical features.

Pathological data	Patients Number 39 (%)	ALDH1A1		CD44		OCT3/4		ABCG2	
		High Expression	Low Expression	High Expression	Low Expression	High Expression	Low Expression	High Expression	Low Expression
Age									
≤ 40 years	27 (69%)	24(89%)	3(11%)	4(21%)	23(79%)	16(59%)	13(41%)	22(76%)	5(34%)
≥ 40 years	12 (31%)	8(66%)	4(36%)	8(67%)	4(33%)	5(43%)	7(57%)	7(58%)	5(42%)

P Value		*0.15*		*0.51*		*0.88*		*0.33*	

Gender									
Males	9 (23%)	3(33%)	6(67%)	5(55%)	4(45%)	2(22%)	7(78%)	3 (33%)	6(67%)
Females	30 (77%)	23(77%)	7(23%)	22(73%)	8(37%)	19(63%)	11(37%)	21(70%)	9(30%)

P Value		*0.001*		*0.05*		*0.013*		*0.02*	

Tumor size									
PT1	25(64% )	14(56%)	11(44% )	12 (48% )	13(52%)	11(48%)	14 (52% )	15 (60% )	10(40%)
PT2	8(20%)	5(62%)	3(38%)	6 (75%)	2 (25%)	4(50%)	4 (50%)	5 (63%)	3 (37%)
PT3	6(16%)	5(83%)	1(17%)	6 (100%)	0 (0.0%)	4(67%)	2 (33%)	5 (83%)	1(13%)

P Value		*0.02*		* 0.035*		* 0.043*		*0.04*	

Tumor stage									
Stage I	25 (64%)	10 (40%)	15 (60% )	11 (44%)	14 (56%)	12(48%)	13 (52%)	10 (40%)	15 60%)
Stage II	8 (21%)	5 (63%)	3(37%)	6 (75%)	2 (25%)	6(75%)	2 (25%)	5 (62%)	3(38%)
Stage III	6 (15%)	5(83%)	1 (17%)	6 (100%)	0 (0.0%)	5(83%)	1 (17% )	5 (83% )	1(17%)

P Value		*0.01*		* 0.005*		* 0.015*		*0.001*	

## Data Availability

The data used to support the findings of this study are available from the corresponding author upon request.
